# Altered Brain Structure and Functional Connectivity of Primary Visual Cortex in Optic Neuritis

**DOI:** 10.3389/fnhum.2018.00473

**Published:** 2018-12-20

**Authors:** Jing Huang, Yunyun Duan, Sidong Liu, Peipeng Liang, Zhuoqiong Ren, Yang Gao, Yaou Liu, Xiaojun Zhang, Jie Lu, Kuncheng Li

**Affiliations:** ^1^Department of Radiology, Xuanwu Hospital, Capital Medical University, Beijing, China; ^2^Beijing Key Laboratory of Magnetic Resonance Imaging and Brain Informatics, Capital Medical University, Beijing, China; ^3^Department of Radiology, Beijing Tiantan Hospital, Capital Medical University, Beijing, China; ^4^Brain and Mind Centre, Sydney Medical School Nepean, The University of Sydney, Sydney, NSW, Australia; ^5^School of Instrumentation Science and Opto-electronics Engineering, Beihang University, Beijing, China; ^6^Department of Neurology, Tongren Hospital, Capital Medical University, Beijing, China; ^7^Department of Nuclear Medicine, Xuanwu Hospital, Capital Medical University, Beijing, China

**Keywords:** optic neuritis, resting state fMRI, functional connectivity, VBM, MRI

## Abstract

Previous studies have revealed brain adaptations to injury that occurs in optic neuritis (ON); however, the mechanisms underlying the functional connectivity (FC) and gray matter volume (GMV) changes in ON have not been clarified. Here, 51 single attack ON patients and 45 recurrent attacks ON patients were examined using structural MRI and resting-state functional MRI (RS-fMRI), and compared to 49 age- and gender-matched healthy controls (HC). FC analysis with a seed in primary visual cortex (V1 area) was used to assess the differences among three groups. Whole brain GMV was assessed using voxel-based morphometry (VBM). Correlation analyses were performed between FC results, structural MRI and clinical variables. We found positive correlations between the Paced Auditory Serial Addition Test (PASAT) score and FC in V1 area with bilateral middle frontal gyrus. Disease duration is significantly negatively related to FC in V1 area with the left inferior parietal lobule. Compared to the HC, single attack ON patients were found to have decreased FC values in the frontal, temporal lobes, right inferior occipital gyrus, right insula, right inferior parietal lobule, and significant increased FC values in the left thalamus. Recurrent attacks ON patients had the same pattern with single attack ON. No significant differences were found in brain GMV among three groups. This study provides the imaging evidence that impairment and compensation of V1 area connectivity coexist in ON patients, and provides important insights into the underlying neural mechanisms of ON.

## Introduction

Optic neuritis (ON) is an inflammatory demyelination disease of optic nerves, which is the leading cause of vision loss in young adults (Pau et al., 2011). Typical clinical manifestations of ON include sub-acute monocular visual loss, pain during eye movement, and visual field defects. Patients diagnosed with ON have a high risk of multiple sclerosis (MS), and about half of the patients progress to MS within 10 years (Beck et al., 2004). After anti-inflammation treatment, many ON patients can experience visual loss recovery and their conditions become relatively stationary. However, many other ON patients may experience recurrent symptoms with persistent vision loss. The spontaneous vision recovery in ON patients could be due to diminishing inflammation and remyelination, and may depend on individuals’ capability for cortical and sub-cortical visual pathway neuronal plasticity, which was evidenced in previous fMRI studies (Naismith et al., 2009; Mascioli et al., 2012). Functional connectivity (FC) in resting state functional MRI (RS-fMRI) can reflect the correlation of the blood-oxygen-level dependent (BOLD) signals in different brain areas in a time sequence. One of the most common FC analysis methods is the seed correlation analysis. Voxel-based morphometry (VBM) is a whole-brain morphology analysis method which compares the voxel-wise, intra-group differences in local brain morphology (Ashburner and Friston, 2000). The VBM and FC methods have been widely used in the studies of inflammatory demyelination diseases of the central nervous system, such as neuromyelitis optica and multiple sclerosis (Rombouts et al., 1998; Henry et al., 2008; Blanc et al., 2012; Duan et al., 2014). However, the mechanisms underlying the brain activity and volumetric changes in ON were less investigated (Gallo et al., 2012; Huang et al., 2016; Benoliel et al., 2017; Backner et al., 2018). We hypothesized that there might be cortical visual pathway changes after the demyelination of optic nerve in ON patients. In this study, we combined the VBM method with the seed correlation method to explore the primary visual cortex (V1 area) and the whole brain FC changes in patients with single and recurrent attacks ON, (1) assessing the differences in gray matter volume (GMV) and FC between the three groups, (2) exploring the correlation between the FC measurements and the clinical assessments, and the structural MRI variables, and (3) investigating the mechanisms underlying the FC changes in single and recurrent attacks ON patients. To our best knowledge, this study represents the first attempt to explore the changes in brain structure and FC in two types of ON patients.

## Materials and Methods

### Subjects

Fifty-one single attack ON patients (9 males, 42 females; mean age 37.51 ± 13.03 years) and 45 recurrent attacks ON patients (13 males, 32 females; mean age 33.80 ± 13.93 years) were included in this study, selected using the following criteria: (1) vision loss with or without eye pain; (2) visual field defects associated with damage to nerve fibers; (3) exclusion of other possible diagnoses, such as ischemic, toxic, genetic, metabolic, or invasive optic neuropathy; (4) not been treated with medications (e.g., corticosteroids) within 4 weeks before MRI scanning; (5) sufficient image quality. There was no limit for disease duration, number of ON attacks, or side for ON. Patients were recruited from the Xuanwu Hospital, Beijing, China. All patients underwent a baseline clinical MRI, no patients were found having brain lesions. Healthy controls included 49 age- and gender-matched subjects (HCs, 14 males, 35 females; mean age 33.57 ± 10.63 years) who met the following inclusion criteria: (1) no neurological or psychiatric disorders; (2) no visual system related diseases; (3) no abnormality in brain MRI scans. The clinical assessments, including disease duration, 3-s paced auditory serial addition test (PASAT-3) and 2-s PASAT (PASAT-2) of the patients were recorded by an experienced neurologist (JY). This study was approved by the institutional review board of Xuanwu Hospital, and all subjects gave written informed consent.

### MRI Acquisition

The MRI data were acquired on a 3.0-T MR system (Trio Tim; Siemens, Erlangen, Germany) with a 12-channel head coil. Resting-state functional MR imaging data were collected using an echo planar imaging sequence, with 35 axial sections acquired; TR/TE = 2000/30 ms; flip angle = 90°; slice thickness = 3 mm; gap = 1 mm; in-plane resolution, 3.5 mm × 3.5 mm; and matrix size = 64 × 64. During resting-state fMRI, subjects were instructed to keep their eyes closed, to remain motion-less, and not to think of anything. High resolution T1-weighted images were acquired with a Magnetization Prepared Rapid Acquisition Gradient-Echo (MP-RAGE) sequence (TR/TE = 1600/2.13 ms, TI = 1000 ms, flip angle = 9°, FOV = 256 mm × 224 mm, matrix size = 256 × 224, slice thickness = 1.0 mm, voxel dimensions = 1.0 mm × 1.0 mm × 1.0 mm). The routine sequences, including axial T2-weighted Turbo Spin Echo (TSE, TR/TE = 5000/87 ms, number of signals acquired = 1, echo train length = 15, matrix size = 256 × 256) and axial Fluid-Attenuated Inversion Recovery (FLAIR, TR/TE = 8500/87 ms, TI = 2500 ms, number of signals acquired = 1, matrix size = 256 × 256), were used to exclude subjects with brain lesions. All the routine axial slices were positioned parallel to a line that joins the inferoanterior and inferoposterior parts of the corpus callosum, with an identical FOV (256 mm × 256 mm), number of slices (35) and section thickness (4 mm).

### Image Analysis

#### Data Preprocessing

fMRI Image preprocessing was carried out using Data Processing and Analysis for (Resting-State) Brain Imaging (DPABI) (Yan et al., 2016^1^), which is a toolbox for data processing based on Statistical Parametric Mapping (SPM8^2^). The first 10 volumes of the functional images were discarded to reach signal equilibrium and allow participants to adapt to the scanning noise. fMRI images were then corrected for within-scan acquisition time differences between slices and then realigned to the first volume to correct for inter-scan head motions. No participant had head motion of more than 1.5 mm displacement in any of the x, y, or z directions, or 1.5° of any angular motion throughout the course of the scan. We spatially normalized the realigned images to the standard echo-planar imaging template and resampled them to 3 mm × 3 mm × 3 mm. The normalized images were then spatially smoothed with a Gaussian kernel of 8 mm × 8 mm × 8 mm full-width half-maximum (FWHM) to decrease spatial noise. To further reduce the effects of confounding factors, we used linear regression process to remove the effects of head motion and other possible nuisance variables: 24 motion parameters, WM and CSF signal. Temporal filtering (0.01–0.08 Hz) was applied to reduce the effect of low-frequency drifts and high-frequency noise.

#### FC Analysis

We used the V1 area as the seed region to calculate FC by using DPABI. The V1 area of the brain generally refers to brodmann area 17 (BA 17), as region of interests (ROIs) using the software WFU Pick Atlas^3^, which has been used in previous studies (Yu et al., 2008; Ding et al., 2013; Zhu et al., 2018). The centers of the ROIs were (-8, -76, 10) and (7, -76, 10) in Montreal Neurological Institute coordinates. For each subject, we produced the correlation map by computing the correlation coefficients between the time course of the seed and all other brain voxels. Correlation coefficients were normally transformed to *z*-values using Fisher’s r-to-z transform (Lu et al., 2007; Liu et al., 2017).

#### Voxel-Based Morphometry

A VBM analysis was performed with VBM8 toolbox^4^ in SPM8 (see text footnote 2). Images were segmented into GM, white matter (WM), and cerebrospinal fluid (CSF) by using the Diffeomorphic Anatomical Registration Through Exponentiated Lie algebra (DARTEL) algorithm (Ashburner, 2007). Following segmentation, the images underwent non-linear normalization to MNI space using the DARTEL algorithm. The images were modulated by non-linear warping only. Finally, the resulting GM images were smoothed with an 8 mm FWHM isotropic Gaussian Kernel.

### Statistical Analyses

To identify the regions that showed significant differences in connectivity to the V1 area among the three groups, a one-way analysis of variance (ANOVA) (*p* < 0.05, AlphaSim corrected, single voxel threshold of *p* < 0.001 and cluster size >13 voxels) followed by *post hoc t*-tests were employed between the three groups. In *post hoc t*-tests, the resulting statistical maps were corrected for multiple comparisons to a significant level of *p* < 0.05 by combining single voxel threshold of *p* < 0.001 and cluster size >39 voxels (single attack ON and HC), >38 voxels (recurrent attacks ON and HC), and >22 voxels (single attack ON and recurrent attacks ON) by using AlphaSim program. In VBM analysis, the statistical threshold was set at *p* < 0.05, by combining single voxel threshold of *p* < 0.001 and cluster size >27 voxels which using the AlphaSim program, within the GM mask. Correlation analysis was performed to explore the relationships between FC changes and clinical variables (disease duration, PASAT and visual acuity) by using DPABI (*p* < 0.05, AlphaSim corrected, by combining single voxel threshold of *p* < 0.001 and cluster size >17 voxels).

## Results

### Demographic, Clinical Characteristics

Table 1 shows the demographic and clinical (disease duration, PASAT and visual acuity) information of the subject groups. There were no significant differences in gender and age, between the three groups, but the differences in disease duration, PASAT and visual acuity were significant (*p* < 0.01) between the two ON groups.

**Table 1:** Demographics and clinical characteristics of all participants.

Characteristics	Single attack ON patients (*n* = 51)	Recurrent ON patients (*n* = 45)	Healthy controls (*n* = 49)	*p-*Values
Mean age (range) (years)	37.51 (15–60)	33.80 (24–58)	33.58 (21–59)	0.86^a^
Gender (M/F)	9/42	13/32	14/35	0.367^b^
Median PASAT-3	45.0 ± 10.5	44.6 ± 9.8	58.2 ± 8.4	<0.001^a^
Median PASAT-2	38.2 ± 11.2	33.8 ± 10.7	46.2 ± 8.5	0.002^a^
Median disease duration (range) (months)	1.3 (0.2–4)	50.4 (38.4–96)	–	<0.0001^c^
Log MAR scores	0.92 ± 0.95	0.70 ± 0.90	–	0.48^c^


*PASAT, Paced Auditory Serial Addition Test; ^*a*^Main effect of group in ANOVA; ^*b*^Chi-squared test; ^*c*^Two-sample *t*-test.*

### Differences in Functional Connectivity

**FIGURE 1:**
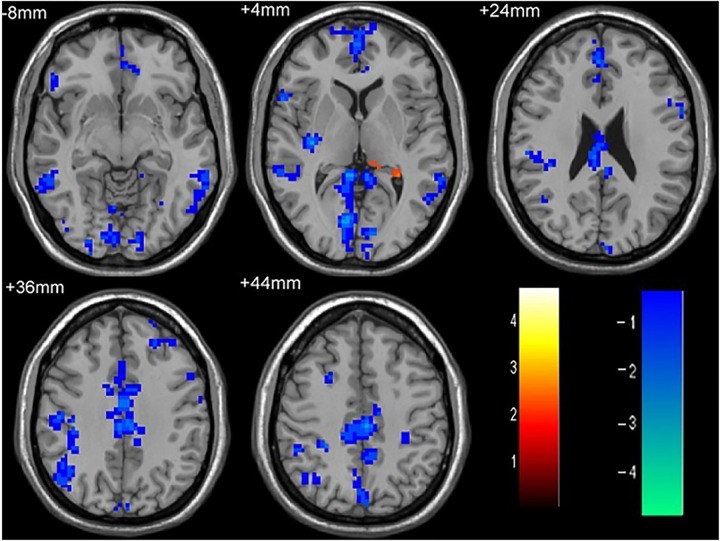
The differences in FC between single attack ON patients and HC subjects. Blue represents the regions with decreased FC values in the bilateral middle temporal gyrus, left medial cingulum, right inferior occipital gyrus, right insula, right middle frontal gyrus, right inferior frontal gyrus, left superior temporal gyrus, right inferior parietal lobule. Red represents the regions with increased FC values in the left thalamus in single attack ON patients (corrected *p* < 0.05 using AlphaSim).

**FIGURE 2:**
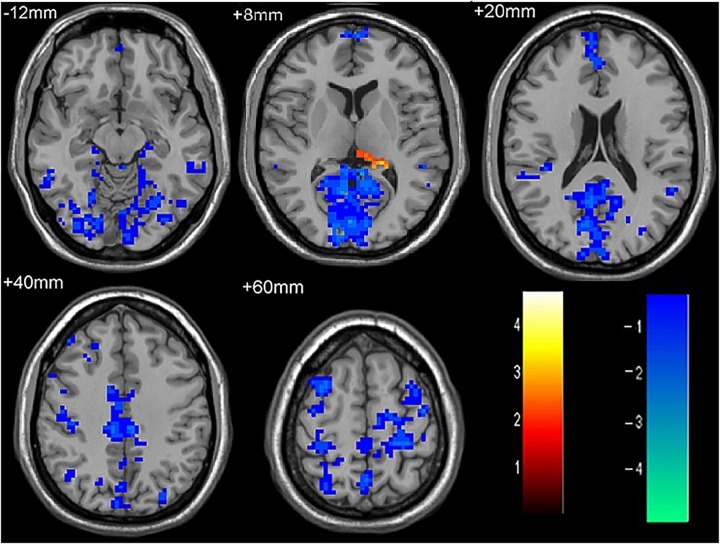
The differences in FC between recurrent attacks ON patients and HC subjects. Blue represents the regions where recurrent attacks ON patients showed decreased FC values in the left middle frontal gyrus, left lingual gyrus, left middle temporal gyrus, left superior temporal gyrus, left medial occipital, left cingulate gyrus, right middle frontal gyrus, right inferior frontal gyrus, right superior parietal lobule. Red represents the regions that recurrent attacks ON increased FC values in the left thalamus (corrected *p* < 0.05 using AlphaSim).

**FIGURE 3:**
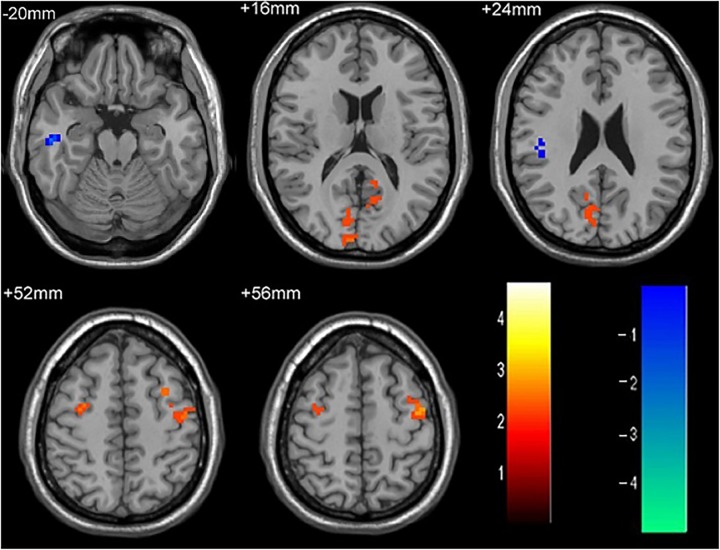
The differences in FC between single attack ON patients and recurrent attacks ON patients. Blue represents the regions that recurrent attacks ON decreased FC values in the right middle temporal gyrus, right middle frontal gyrus. Red represents the regions that recurrent attacks ON increased FC values in the right inferior frontal gyrus, left middle frontal gyrus, right middle occipital gyrus, right inferior parietal lobule (corrected *p* < 0.05 using AlphaSim).

As shown in Figure 1 and Table 2, compared to the HC group, patients with single attack ON were found having decreased values of FC in bilateral middle temporal gyrus, left medial cingulum, right inferior occipital gyrus, right insula, right middle frontal gyrus, right inferior frontal gyrus, left superior temporal gyrus, right inferior parietal lobule, and significantly increased values of FC in the left thalamus. Figure 2 and Table 3 show that patients with recurrent attacks ON were found having decreased FC values in left middle frontal gyrus, left lingual gyrus, left middle temporal gyrus, left superior temporal gyrus, left medial occipital, left cingulate gyrus, right middle frontal gyrus, right inferior frontal gyrus, right superior parietal lobule, and increased FC values in the left thalamus. Compared with single attack ON patients, recurrent attacks ON patients showed decreased FC values in the right middle temporal gyrus, right middle frontal gyrus, and increased FC values in the right inferior frontal gyrus, left medial frontal gyrus, right middle occipital gyrus, right inferior parietal lobule, as shown in Figure 3 and Table 4.

**Table 2:** Results of the FC with the primary visual area in the single attack ON patients and the HC subjects.

Anatomic area	MNI coordinates			Cluster size	*T*-score
	*x*	*y*	*z*		
Left thalamus	-21	-39	9	48	-3.303
Right middle temporal gyrus	60	-18	-33	107	3.84
Left middle temporal gyrus	-39	-54	-27	117	3.5773
Left medial cingulum	-24	-33	29	216	4.2613
Right inferior occipital gyrus	42	-84	-15	161	3.9082
Right insula	36	-18	6	52	4.0606
Right middle frontal gyrus	3	69	9	281	4.7187
Right inferior frontal gyrus	60	15	3	47	3.1109
Left superior temporal gyrus	-60	-24	12	49	3.2109
Right inferior parietal lobule	39	-33	18	105	3.5221


*HC-single attack ON patients (corrected *p* < 0.05 using AlphaSim). *T*-score > 0, single attack ON’s brain regions with less functional activity than HC; *T*-score < 0, single attack ON’s brain regions with more functional connectivity than HC.*

**Table 3:** Results of the FC with the primary visual area in the recurrent attacks ON patients and the HC subjects.

Anatomic area	MNI coordinates			Cluster size	*T*-score
	*x*	*y*	*z*		
Left thalamus	-15	-24	-51	87	-3.7806
Left middle frontal gyrus	-6	69	6	230	3.6814
Left lingual gyrus	12	-90	9	404	4.9704
Left middle temporal gyrus	-57	-30	-21	55	2.9477
Left superior temporal gyrus	-54	-48	15	76	3.3748
Left medial occipital	-30	-81	36	64	3.3309
Left cingulate gyrus	-21	-27	54	301	4.033
Right middle frontal gyrus	24	36	36	117	3.2447
Right inferior frontal gyrus	33	6	60	130	3.5226
Right superior parietal lobule	42	-60	33	133	3.2135


*HC-recurrent attacks ON patients (corrected *p* < 0.05 using AlphaSim). *T*-score > 0, recurrent attacks ON’s brain regions with less functional activity than HC; *T*-score < 0, recurrent attacks ON’s brain regions with more functional connectivity than HC.*

**Table 4:** Results of the FC with the primary visual area in the single attack and the recurrent attacks ON patients.

Anatomic area	MNI coordinates			Cluster size	*T*-score
	*x*	*y*	*z*		
Right middle temporal gyrus	48	9	-36	28	3.3476
Right middle frontal gyrus	36	27	36	45	3.4806
Right inferior frontal gyrus	18	24	-18	44	-3.4187
Left middle frontal gyrus	-12	27	-9	31	-3.1665
Right middle occipital gyrus	9	-96	15	29	-2.8683
Right inferior parietal lobule	45	-33	30	41	-3.1209


Single attack ON patients-recurrent attacks ON patients (corrected *p* < 0.05 using AlphaSim). *T*-score > 0, recurrent attacks ON’s brain regions with less functional activity than single attack ON; *T*-score < 0, recurrent attacks ON’s brain regions with more functional connectivity than single attack ON.

### Differences in Gray Matter Volume

No differences in brain GMV were found between the single attack ON, recurrent attacks ON and HC groups (corrected *p* < 0.05 using AlphaSim).

### Correlation Analysis

In the two groups of ON patients, we examined the correlations between disease duration, PASAT, visual acuity and the extent of altered V1 area FC. We observed significant positive correlations between the PASAT-2/PASAT-3 and FC in V1 area with bilateral middle frontal gyrus in ON patients. Disease duration is significant negatively correlated with FC in V1 area in the left inferior parietal lobule.

## Discussion

In this study, we explored the differences in GMV and FC between ON patients and HC using both VBM and RS-FC methods. To our knowledge, this study represents the first study that demonstrates visual cortical adaptations in primary visual cortex using fMRI in both single and recurrent attacks ON patients. The results of VBM analysis showed no difference in GMV between the three groups. Furthermore, we found patients with single attack and recurrent attacks ON had significantly increased FC with the primary visual area only in the left thalamus. These findings suggest that thalamus which is a central hub in the brain may play important roles in FC in single attack ON patients. The thalamus is an important relay of motor and sensory information to and from the cerebral cortex; structural and functional thalamic alterations had been widely reported in patients with clinically isolated syndrome (CIS) and MS in both task-based and resting state functional MRI studies. These findings suggest increased connectivity in the thalamus could reflect the same disease-associated functional change with the demyelination disease. While we demonstrate that in single attack ON patients there are significant decreased FC in the bilateral middle temporal gyrus, left medial cingulum, right inferior occipital gyrus, right insula, right medial frontal gyrus, right inferior frontal gyrus, left superior temporal gyrus, right inferior parietal lobule, these brain regions are commonly referred to as the default mode network (DMN), which suggests that when ON patients suffer from visual impairment, their default networks may have compensatory activities. Roosendaal et al. (2010) demonstrated that brain remodeling occurs mainly in the early stage of MS (CIS), and stopped in relapse remission MS stage, suggesting that brain remodeling decreases with MS disease progression, yet the genesis of the specific brain remodeling is not clear. Our findings suggest that in the first onset of ON, there is an abnormal reduction of FC in the visual related brain regions with concomitant compensatory activities in DMN, which provides important insights to the mechanism of the early brain damage, caused by the disease. Compared with the single attack ON patients, recurrent attacks ON patients showed decreased FC with the primary visual area in the left medial frontal gyrus, left lingual gyrus, left medial temporal gyrus, left superior temporal gyrus, left medial occipital, left cingulate gyrus, right middle frontal gyrus, right inferior frontal gyrus, right superior parietal lobule. The overwhelming number of regions with decreased FC suggests that functional reorganization may not be able to compensate the functional damage in the recurrent episodes of the disease.

Interestingly, we found the right inferior parietal lobule was involved in both decreased and increased FC with the primary visual area. This may be due to the inferior parietal lobule containing multiple anatomical landmark based brain regions (Tzourio-Mazoyer et al., 2002), but current analysis cannot isolate these tiny brain regions. The decreased connectivity between the primary visual area and the inferior parietal lobule was found in many diseases, such as ON (Mascioli et al., 2012), MS (Liu et al., 2015), amblyopia (Ding et al., 2013), and epilepsy (Lv et al., 2014). The right inferior parietal lobule is predominantly known to be involved in spatial attention (Cicek et al., 2007; Husain and Nachev, 2007; Singh-Curry and Husain, 2009), and thus, we hypothesize that the visual abnormality in recurrent attacks ON patients may indicate they have the visual-spatial processing deficits. Further study with standard visual-spatial processing tests will be warranted to confirm this hypothesis. In a previous study of the VEP components in ON (Andersson and Siden, 1995), Andersson et al. found that the inferior parietal lobule contributes to visual word recognition. Huang et al. (2016) found increased white matter volumes in the left inferior parietal lobule in the ON patients, which may reflect a compensation effect by functional reorganization for the damaged brain tissues in ON. In this study, our results are in line with these findings from the perspective of FC in RS-fMRI. No significant differences in the GMV was found between the single attack ON, recurrent attacks ON and HC groups, which indicate that functional compensation may occurs prior to the structural changes, and imply a dissociation between structural damage and functional impairment in these diseases.

Paced Auditory Serial Addition Test is a measure for complex attention, executive function, working memory and information-processing. A few studies (Audoin et al., 2005; Summers et al., 2008) used PASAT to evaluate cognitive performance in CIS patients, and found reduced information processing speed among them. Our results are in line with the previous findings, and confirm the existence of cognitive deficits in ON type CIS patients. Furthermore, we examined the relationships of FC alterations and clinical variables in ON patients, and found positive correlations between the PASAT-2/PASAT-3 and FC in V1 area with bilateral middle frontal gyrus in ON patients. Disease duration is negatively correlated with FC in V1 area and left inferior parietal lobule in ON patients. As demonstrated in previous study (Seeley et al., 2007), middle frontal gyrus was implicated in the salience network, has often been involved in cognitive; therefore our finding may suggest that cerebellum plays a fundamental part in the cognition deficit in ON patients. This point will need to be further investigated. Large-scale and longitudinal studies correlating cognitive performance with advanced MRI measures will be required to better understand the cognitive impairment caused by ON.

There are some limitations in this study. First, this study is a preliminary cross-sectional study; further longitudinal study is warranted to investigate the reproducibility of the FC abnormalities in ON patients. Second, our current method cannot segment the sub-anatomical regions within the visual area for VBM and FC analysis, for instance the lateral geniculate nucleus which is a group of important second-order neurons; therefore limit the analysis of the abnormality in the visual pathway. Third, this study had a relatively small sample-size (145 subjects) and a limited number of structural and functional vision measures [e.g., optical coherence tomography (OCT), high- and low-contrast letter acuity testing, and visual fields and quality-of-life measures (VFQ-25)]. Further studies with a larger sample size and comprehensive clinical data are warranted to understand the correlations between FC abnormalities and clinical metrics.

## Conclusion

In summary, we demonstrated evidence of cortical neuroplasticity in both single and recurrent attacks ON patients by using RS-fMRI, and the impairment and compensation of V1 area connectivity coexist in these patients. These findings provide important insights into the underlying neural mechanisms of ON.

## Author Contributions

JH, JL, and KL designed the experiments and wrote the manuscript. JH, YD, XZ, and ZR carried out the experiments. YG, SL, and PL analyzed the experimental results. YL and PL analyzed the sequencing data and developed the analysis tools.

## Conflict of Interest Statement

The authors declare that the research was conducted in the absence of any commercial or financial relationships that could be construed as a potential conflict of interest.

## Footnotes

1 http://rfmri.org/DPABI

2 http://www.fil.ion.ucl.ac.uk/spm

3 http://www.ansir.wfubmc.edu/

4 http://dbm.neuro.uni-jena.de/vbm
